# Anti-Thrombotic Effects of Artesunate through Regulation of cAMP and PI3K/MAPK Pathway on Human Platelets

**DOI:** 10.3390/ijms23031586

**Published:** 2022-01-29

**Authors:** Shin-Sook Yoon, Hyuk-Woo Kwon, Jung-Hae Shin, Man Hee Rhee, Chang-Eun Park, Dong-Ha Lee

**Affiliations:** 1 Department of Biomedical Laboratory Science, Namseoul University, Cheonan 31020, Korea; yss28@hanmail.net (S.-S.Y.); pce@nsu.ac.kr (C.-E.P.); 2 Department of Biomedical Laboratory Science, Far East University, Eumseong 27601, Korea; hyuk-woo@hanmail.net; 3 Department of Veterinary Medicine, College of Veterinary Medicine, Kyungpook National University, Daegu 41566, Korea; mlsjshin@naver.com (J.-H.S.); rheemh@knu.ac.kr (M.H.R.); 4 Molecular Diagnostics Research Institute, Namseoul University, Cheonan 31020, Korea

**Keywords:** artesunate, platelet aggregation, cyclic nucleotide, intracellular Ca^2+^, granule secretion

## Abstract

Normal activation of platelets and their aggregation are crucial for proper hemostasis. It appears that excessive or abnormal aggregation of platelets may bring about cardiovascular diseases such as stroke, atherosclerosis, and thrombosis. For this reason, finding a substance that can regulate platelet aggregation or suppress aggregation will aid in the prevention and treatment of cardiovascular diseases. Artesunate is a compound extracted from the plant roots of *Artemisia* or *Scopolia*, and its effects have shown to be promising in areas of anticancer and Alzheimer’s disease. However, the role and mechanisms by which artesunate affects the aggregation of platelets and the formation of a thrombus are currently not understood. This study examines the ways artesunate affects the aggregation of platelets and the formation of a thrombus on platelets induced by U46619. As a result, cyclic adenosine monophosphate (cAMP) and cyclic guanosine monophosphate (cGMP) production were increased significantly by artesunate relative to the doses, as well as phosphorylated vasodilator-stimulated phosphoprotein (VASP) and inositol 1,4,5-trisphosphate receptor (IP_3_R), substrates to cAMP-dependent kinase and cGMP-dependent kinase, in a significant manner. The Ca^2+^, normally mobilized from the dense tubular system, was inhibited due to IP_3_R phosphorylation from artesunate, and phosphorylated VASP aided in inhibiting platelet activity via αIIb/β_3_ platelet membrane inactivation and inhibiting fibrinogen binding. In addition, MAPK and PI3K/Akt phosphorylation was inhibited via artesunate in a significant manner, causing the production of TXA_2_ and intracellular granular secretion (serotonin and ATP release) to be reduced. Therefore, we suggest that artesunate has value as a substance that inhibits platelet aggregation and thrombus formation through an antiplatelet mechanism.

## 1. Introduction

Worldwide, the most prevalent cause of death is cardiovascular disease (CVD). The United States reported that one in seven deaths were due to coronary heart disease and one in nine deaths were due to heart failure [1]. The role of platelets is important in the formation of blood clots to maintain hemostasis after blood vessel damage and to prevent blood loss. However, excessive activation and aggregation of platelets is a major cause of thrombus formation that causes cardiovascular diseases such as coronary artery disease, atherosclerosis, heart failure, and stroke [2].

Thrombotic events are dramatically lowered through the inhibition of platelets via pharmacological agents, and CVD treatment and prevention uses such clinical drugs. Unfortunately, complications and side effects such as gastric bleeding, aplastic anemia, or even thrombocytopenia can arise from these drugs, often minimizing the benefits. Therefore, they require the development of minimal prophylactic or therapeutic alternatives [3]. In addition to antiplatelet drug-based treatment options for drug-related complications, problems related to CVD, and disorders related to thrombosis, the use of natural products is generating a great deal of interest due to the natural bioactive compounds along with their applications for medical treatment of CVD prevention and treatment [4]. Similarly, many products that are natural, such as traditional diets from the Mediterranean and plants with medicinal purposes, have shown antiplatelet and cardioprotective properties in the prevention (primary and secondary) of CVD [5,6,7].

The antiplatelet agents, theophylline and Verapamil, reduce the amount of [Ca^2+^]_i_ needed for proper platelet aggregation via an increase in cyclic adenosine monophosphate (cAMP) levels [8]. In addition, cyclic guanosine monophosphate (cGMP) levels in platelets are increased with the use of vasodilators (nitroprusside and molsidomine) and cGMP phosphodiesterase (PDE) inhibitors (zaprinast and erythro-9- (2-hydroxy-3-nonyl) adenine) [9]. Prostaglandin I_2_ and nitric oxide are released from vascular endothelial cells during normal blood circulation, causing platelets to produce cAMP and cGMP. Increasing cAMP and cGMP levels cause protein kinase A (PKA) and protein kinase G (PKG) activation, respectively. PKG and PKA are known to cause the phosphorylation of the substrate proteins vasodilator-stimulated phosphoprotein (VASP) and inositol 1, 4, 5-trisphosphate receptor (IP_3_R) [10]. Phosphorylation of IP_3_R inhibits Ca^2+^ recruitment into the cytoplasm from the dense tubular system by inactivating IP_3_R [11]. In platelets, VASP is the major substrate of PKG and PKA and contributes to the regulation of αIIb/β3 activation and the activity of actin filament. When cGMP-dependent VASP Ser239 and cAMP-dependent VASP Ser157 are phosphorylated, the activation of αIIb/β_3_ and the elongation of actin filament are inhibited [12,13]. As a result, IP_3_R, once phosphorylated, can confirm the antiplatelet effect of Ca^2+^ mobilization inhibition, and phosphorylated VASP inhibits platelet activity through αIIb/β_3_ inhibition.

In addition, substances such as ATP and serotonin released through granule secretion are important in platelet aggregation and are known to be involved in phosphorylation of phosphoproteins such as Mitogen-activated protein kinases (MAPK) and PI3K/Akt [14]. The phosphorylating enzymes, MAPK, are called p38 MAPK, c-Jun N-terminal kinase (JNK), and extracellular signal-regulated kinase (ERK). In hemostasis and thrombus formation, the role of MAPK has been thoroughly studied, and they are known to function in intracellular signaling [15]. MAPK is reported to react to multiple agonists and show activity by being phosphorylated, and it is commonly found in human platelets [16,17,18]. This signal transduction molecule, once phosphorylated, is acknowledged to play a crucial part in stimulating platelet granule secretion [19,20]. Furthermore, MAPK is known to phosphorylate cPLA_2_ found in the cell membrane, which then results in an increase in TXA_2_, leading to further platelet activation and aggregation [21,22]. In addition, the PI3K/Akt pathway has been shown to help with platelet function regulation, involving behaviors such as dense granule secretion in platelets and overall platelet aggregation [23]. Therefore, inhibiting phosphorylated PI3K/Akt is helpful for assessing substances or components that exhibit antiplatelet effects.

Artesunate is a semisynthetic derivative of artemisinin among the major active ingredients of Artemisia and is known as a novel antimalarial drug characterized by low toxicity and tolerance [24]. In addition, artesunate showed antitumor activity and was successfully used in the treatment of metastatic melanoma patients in clinical practice [24]. Earlier studies have shown that artesunate increases oxygen free radical production intracellularly and intervenes in the nuclear factor (NF)-κB- and phosphatidylinositol-4,5-bisphosphate 3-kinase/Akt-mediated signaling pathways, producing damage to DNA, and it has been reported to interfere with cell cycle regulation and metastasis [25,26]. Artesunate has been reported to improve patient survival by significantly reducing the amount of stroke (cerebral infarction) and enabling neurological recovery [27]. It has been reported that artesunate reduces the ability of COX-2 to inhibit the growth of human gastric cancer cells and induce apoptosis. COX-2 is an isoenzyme similar in function and form to COX-1, which acts to produce TXA_2_ in platelet metabolism. In fact, NSAID-based drugs such as aspirin, which are widely used as antiplatelet agents, exhibit a function of inhibiting both COX-1 and COX-2 [28]. Additionally, it was reported that artesunate’s analogue artemisinin inhibits inflammation while also inhibiting MAPK pathways [29]. Inhibition of MAPK activity is characteristic of antiplatelet substances, as it is associated with inhibition of platelet secretion [30].

Currently, the role and mechanisms of artesunate, during both platelet activation and platelet aggregation, are yet to be discovered. To bring an understanding to the antiplatelet effect of artesunate, we investigated the effect of artesunate on calcium mobilization and granule secretion by examining the regulation of circulating nucleotides via the regulation of PI3K/Akt and MAPK. Moreover, we explored whether artesunate finally participates in the aggregation of platelets and the formation of a thrombus. If artesunate is demonstrated to be effective as an antiplatelet agent, it is proposed that artesunate could be used to prevent thrombosis-related CVD.

## 2. Results

### 2.1. Effects of Artesunate on U46619-Induced Platelet Aggregation

**Figure 1:**
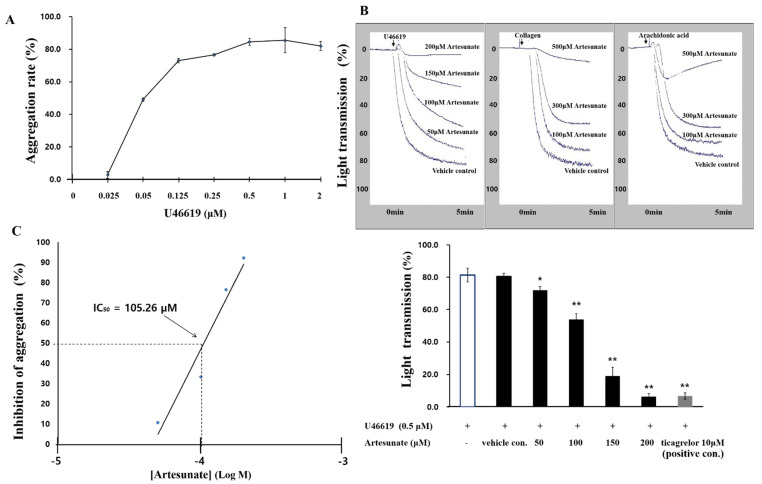
Artesunate’s effect on platelet aggregation induced by U46619. (**A**) The U46619 threshold dose on human platelet aggregation. (**B**) Artesunate’s effect on platelet aggregation. (**C**) IC_50_ value of artesunate on platelet aggregation induced from U46619. The results are shown as mean ± SD (*n* = 4). * *p* < 0.05, ** *p* < 0.001 in comparison to the U46619−induced vehicle control.

The maximum aggregation induction concentration of U46619, a TXA_2_ analogue that induces platelet aggregation, was found to be 0.5 µM, and 0.5 µM U46619 was used to induce aggregation in this experiment (Figure 1A). With platelets mixed with 0.5 µM U46619 and 0.1% DMSO, in order to start aggregation, the rate of aggregation came to 80.5 ± 2.1% (vehicle control), which was not significantly different from the results of induction of aggregation without 0.1% DMSO. When different artesunate concentrations (50 to 200 μM) were added, no cytotoxicity was observed (data not shown). The results showed that aggregation was suppressed by artesunate (Figure 1B). At this moment, the half-maximal inhibitory concentration (IC50) of artesunate came out to 105.26 μM (Figure 1C). As a positive control, the antiplatelet substance ticagrelor (10 μM) was compared with the platelet aggregation inhibitory effect (Figure 1B). We confirmed that artesunate had an inhibitory effect even when aggregation was induced with collagen and arachidonic acid (Figure 1B). However, their inhibitory effect on artesunate was weaker than that induced by U46619. In any case, these results demonstrate that artesunate has a potent platelet aggregation inhibitory effect.

### 2.2. Effects of Artesunate on the Cyclic Nucleotides Production

**Figure 2:**
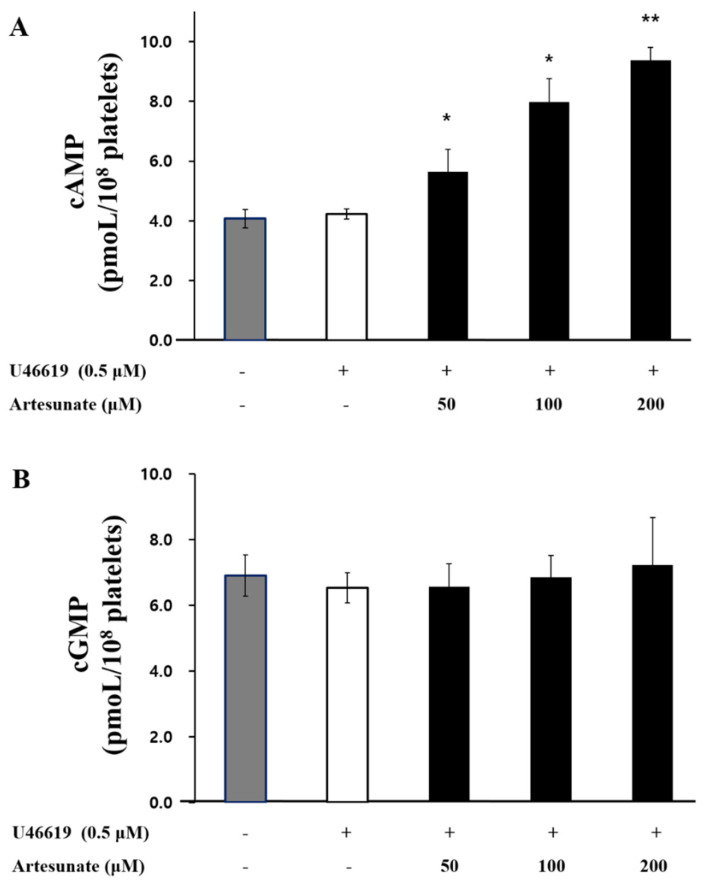
Artesunate’s effect on the production of cyclic nucleotides. (**A**) Effects of artesunate on cAMP production. (**B**) Effects of artesunate on cGMP production. Incubation of the suspended platelets (10^8^ cells/mL) occurred at 37 °C while adding different artesunate concentrations, then 2 mM CaCl_2_ was added with U46619 (0.5 μM) for stimulation at a duration of 5 min. Termination of the reaction occurred by adding 1 M HCl, and the cGMP/cAMP were measured with a cAMP or cGMP EIA kit. The results are shown as mean ± SD (*n* = 4). * *p* < 0.05, ** *p* < 0.001 in comparison to the U46619−induced platelets.

Artesunate’s effect on the production of cGMP or cAMP, which is known to have antagonistic effects in platelet activation, was identified. As a result, as shown in Figure 2A, artesunate significantly increased the production of cAMP from 4.23 ± 0.16 pmoL/10^8^ cells to 9.38 ± 0.42 pmoL/10^8^ cells. Furthermore, cGMP production was not significantly increased by artesunate (Figure 2B). These results show that artesunate inhibits platelet activation by significantly increasing cAMP production, but not cGMP production.

### 2.3. Effects of Artesunate on Intracellular Ca^2+^ Mobilization and IP3R Phosphorylation

**Figure 3:**
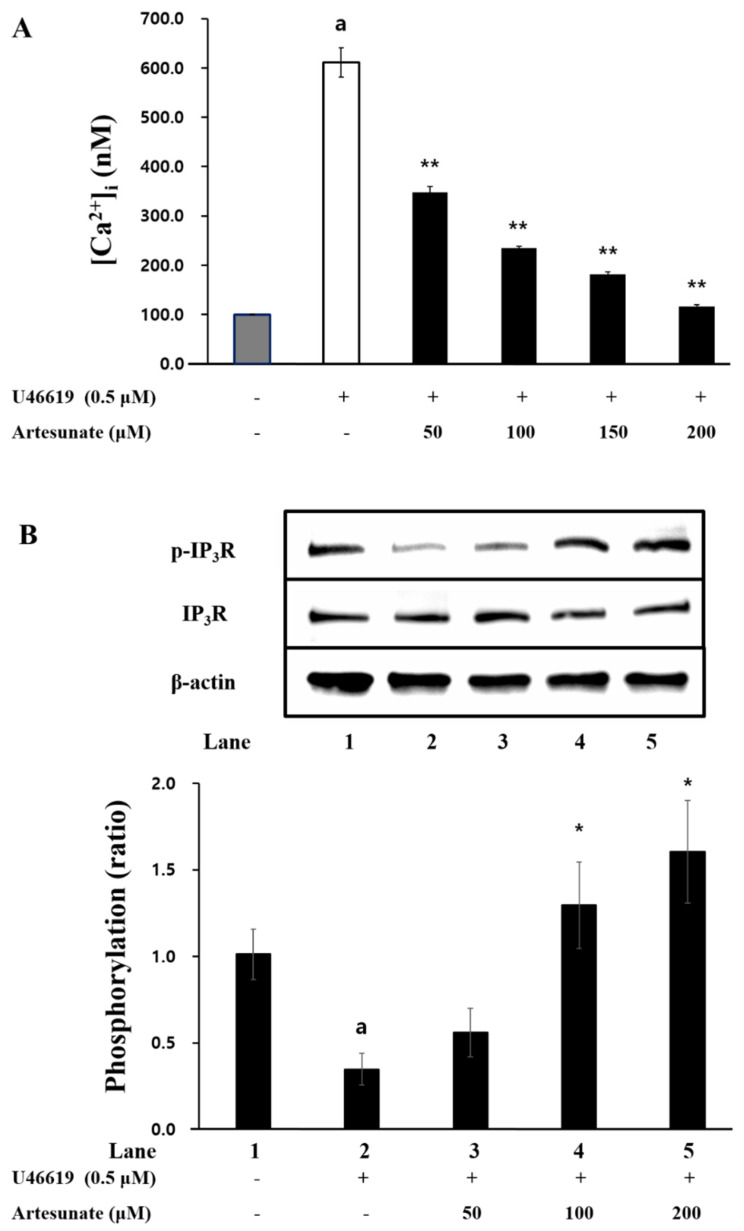
Artesunate’s effect on intracellular Ca^2+^ mobilization and IP_3_R phosphorylation. (**A**) Effects of artesunate on intracellular Ca^2+^ mobilization. (**B**) Effects of artesunate on IP_3_R phosphorylation. For 60 min, incubation of PRP occurred with Fura 2−AM (5 μM) at 37 °C, and the suspending platelets were prepared (10^8^ cells/mL) by the steps specified above. The incubation of washed platelets occurred for 3 min at 37 °C with 2 mM CaCl_2_ and U46619 (0.5 μM) stimulation for a duration of 5 min. A spectrofluorometer from BioTeck Instrument measured the Fura 2 fluorescence. The results are shown as mean ± SD (*n* = 4). ^a^ *p* < 0.05 in comparison to non−stimulated platelets, * *p* < 0.05, ** *p* < 0.001 in comparison to the U46619−induced platelets.

Since intracellular Ca^2+^ mobilization ([Ca^2+^]_i_) is known to be important for platelet activation, this study confirmed artesunate’s effect on [Ca^2+^]_i_. Figure 3A shows [Ca^2+^]_i_ levels were increased from 100.3 ± 0.5 nM to 611.3 ± 29.4 nM by U46619. However, artesunate (50~200 μM) significantly decreased [Ca^2+^]_i_ increased by U46619 (Figure 3A). However, artesunate (50~200 μM) significantly decreased increased [Ca^2+^]i by U46619 (Figure 3A). In addition, this study confirmed artesunate’s effect on IP_3_R phosphorylation, a protein that regulates [Ca^2+^]_i_. As shown in Figure 3B, artesunate (50~200 μM) concentration-dependently increased IP_3_R phosphorylation in U46619-induced platelets. The significance is apparent and confirmed with concentrations above 100 μM. This suggests that the reduction in intracellular Ca^2+^ recruitment by artesunate is due to IP_3_R phosphorylation.

### 2.4. Effects of Artesunate on VASP Phosphorylation and Fibrinogen Binding

**Figure 4:**
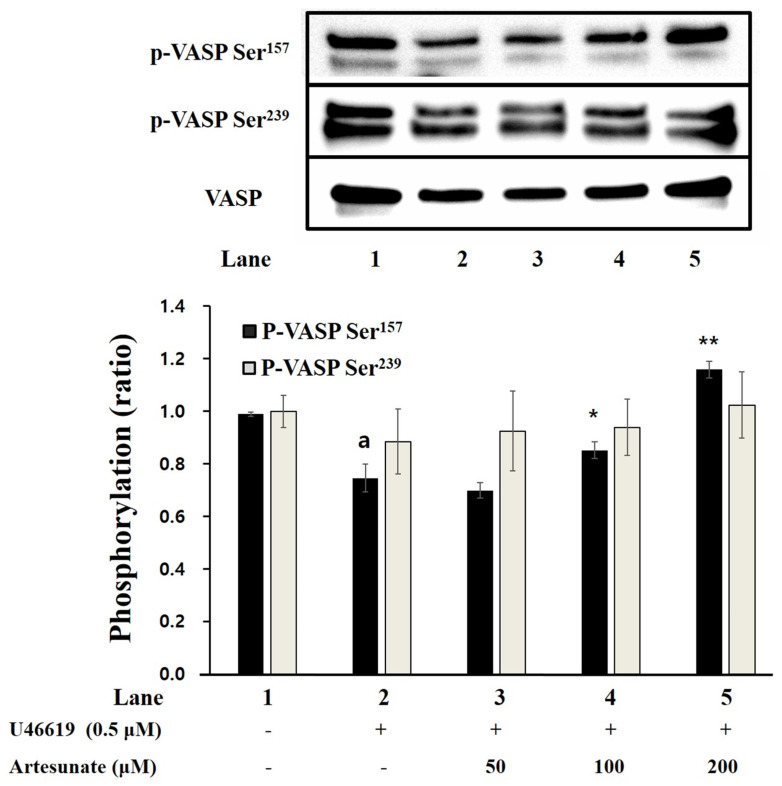
Artesunate’s effect on VASP phosphorylation. Termination of the reaction was carried out using a 1× lysis buffer. A BCA protein kit measured protein concentration from platelet lysates. Isolated protein (20 μg) from 4–20% SDS–PAGE was transferred to the PVDF membrane. The primary antibody was treated with a dilution factor of 1:1000 and the secondary antibody was treated with a dilution factor of 1:2000. The results are shown as mean ± SD (*n* = 4). ^a^ *p* < 0.05 in comparison to non–stimulated platelets, * *p* < 0.05, ** *p* < 0.001 in comparison to the U46619–induced platelets.

As artesunate increased the cAMP production in platelets induced with U46619 (Figure 3), we examined artesunate’s effect on the phosphorylation of cGMP-dependent VASP Ser239 and cAMP-dependent VASP Ser157 in platelets induced with U46619. As shown in Figure 4, VASP Ser157 was significantly increased by artesunate, but VASP Ser239 was not. In particular, the results confirmed that, with 100 μM or more, significance is apparent, which indicates that cAMP production increased by artesunate significantly increased the VASP Ser157 phosphorylation. 

**Figure 5:**
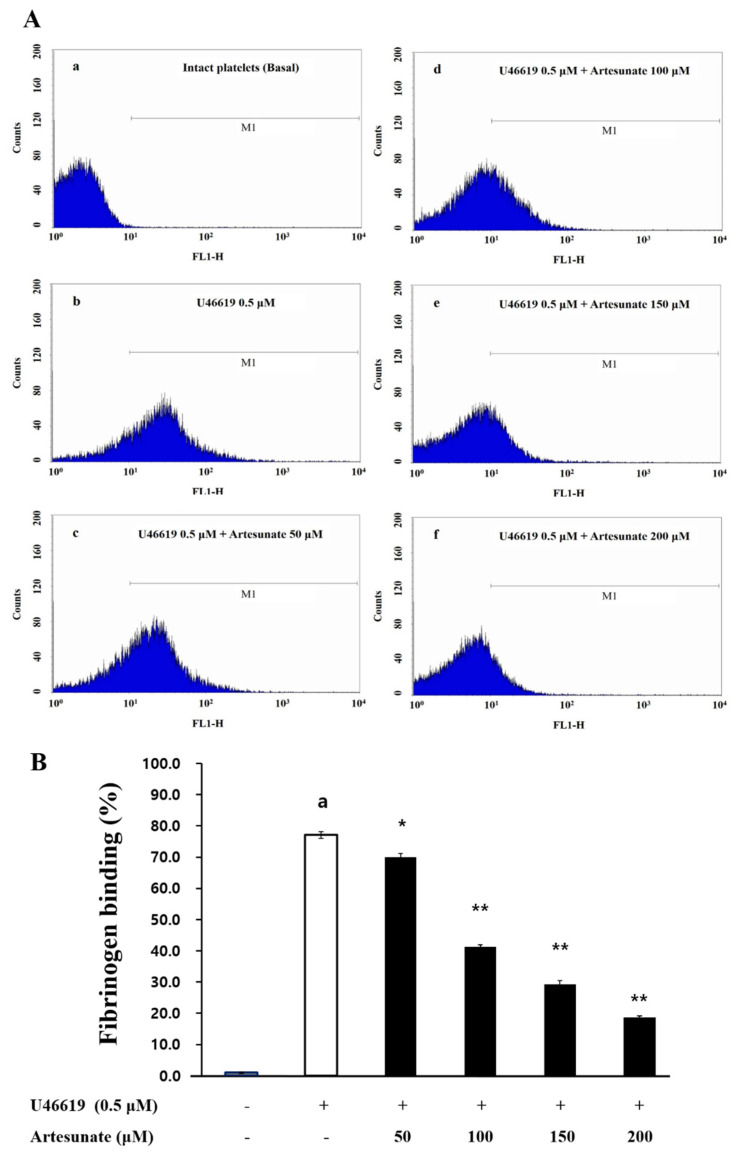
Artesunate’s effects on the binding of fibrinogen. (**A**) The histograms of flow cytometry on the binding of fibrinogen. (**a**) Intact platelets (base); (**b**) U46619 (0.5 μM); (**c**) U46619 (0.5 μM) + artesunate (50 µM); (**d**) U46619 (0.5 μM) + artesunate (100 µM); (**e**) U46619 (0.5 μM) + artesunate (200 µM); (**f**) U46619 (0.5 μM) + artesunate (300 µM). (**B**) Artesunate’s effect on the binding (%) of fibrinogen induced by U46619. After treatment with Alexa Fluor 488-human fibrinogen (30 μg/mL) by adding 2 mM CaCl_2_ to the suspended platelets (10^8^ cells/mL), U46619 (0.5 μM) was used for stimulation at a duration of 5 min. After adding phosphate-buffered saline (PBS, pH 7.4) containing 0.5% paraformaldehyde, the reaction was terminated. Light blocking was used during this process and a flow cytometer (FACS) measured fibrinogen binding. The results are shown as mean ± SD (*n* = 4). ^a^ *p* < 0.05 in comparison to non−stimulated platelets, * *p* < 0.05, ** *p* < 0.001 in comparison to the U46619−induced platelets.

Since VASP Ser157 phosphorylation was increased by artesunate through increased production of cAMP, this study confirmed artesunate’s effect regarding fibrinogen’s binding rate to αIIb/β_3_. Looking at Figure 5A, M1 refers to the cell part that shows fluorescence above the standard, and refers to the cell bound to fibrinogen. Fibrinogen binding to αIIb/β_3_ was increased by U46619 to 77.1 ± 1.1% (Figure 5A(b),B). Yet, the inhibition of fibrinogen binding by artesunate was confirmed in a concentration-dependent manner. Additionally, the confirmed rate of inhibition with artesunate (200 μM) was 75.4% (Figure 5A(f),B).

### 2.5. Effects of Artesunate on TXA_2_ Production and Granule Secretion

**Figure 6:**
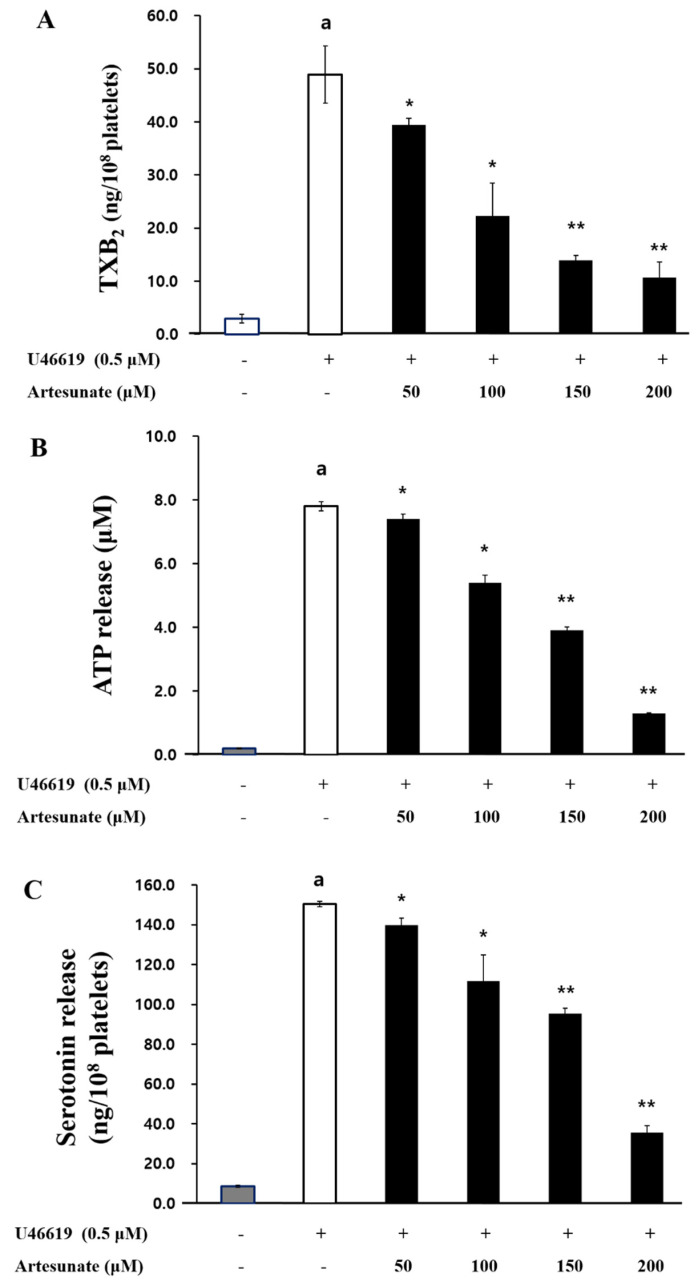
Artesunate’s effect on TXA_2_ production and the secretion of granules. (**A**) Artesunate’s effect on TXA_2_ production. (**B**) Artesunate’s effect on ATP release. (**C**) Artesunate’s effect on the release of serotonin. For 3 min, suspended platelet (10^8^ cells/mL) incubation occurred at 37° with different artesunate concentrations, then 2 mM CaCl_2_ were added to U46619 (0.5 μM) for stimulation at a duration of 5 min. Once the reaction was stopped, the TXA_2_ and granule secretion were measured by each EIA kit. The results are shown as mean ± SD (*n* = 4). ^a^ *p* < 0.05 in comparison to non−stimulated platelets, * *p* < 0.05, ** *p* < 0.001 in comparison to the U46619−induced platelets.

The effect of artesunate on TXA_2_ production, an autacoid that amplifies the aggregation of platelets, was confirmed. As shown in Figure 6A, TXA_2_ production, which was 2.97 ± 0.81 ng/10^8^ cells in intact cells, was increased to 48.93 ± 5.41 ng/10^8^ cells by U46619. However, artesunate (50, 100, 150, and 200 μM) significantly reduced the TXA_2_ production to 39.44 ± 1.28, 22.30 ± 6.20, 13.90 ± 0.89, and 10.70 ± 2.89 ng/10^8^ cells, respectively (Figure 6A).

Artesunate’s effect on ATP and serotonin release concerned with the aggregation of platelets as an index of platelet granule release was confirmed. As a result, U46619 (0.5 μM) increased ATP release from 0.16 ± 0.02 μM in intact cells to 7.81 ± 0.15 μM (Figure 6B); however, the increased ATP release was significantly inhibited by artesunate (50, 100, 150, and 200 μM). In addition, U46619 (0.5 μM) increased serotonin release from 8.65 ± 0.58 ng/10^8^ cells in intact cells to 150.36 ± 1.30 ng/10^8^ cells. Yet, artesunate (50, 100, 150, and 200 μM) reduced the release of serotonin increased by U46619 to 139.70 ± 3.63, 111.87 ± 12.94, 95.39 ± 2.90, and 35.66 ± 3.57 ng/10^8^ cells, respectively (Figure 6C). These results show that artesunate significantly inhibits granule secretion. Since ATP and serotonin are known to be abundant in dense granules, it can be expected that artesunate is related to degranulation in dense granules. However, it is difficult to clearly explain whether artesunate was additionally involved in the degranulation of alpha granules only from the results of this study.

### 2.6. Effects of Artesunate on Phosphorylation of PI3K/Akt and MAPK

**Figure 7:**
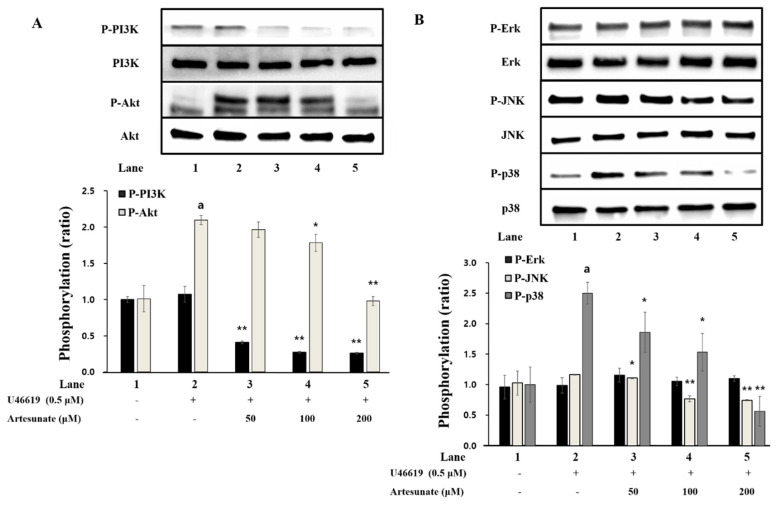
Artesunate’s effect on the phosphorylation of PI3K/Akt and MAPK. (**A**) Artesunate’s effect on PI3K/Akt phosphorylation. (**B**) Effects of artesunate on MAPK phosphorylation. Termination of the reaction was carried out using a 1× lysis buffer. A BCA protein kit measured the protein concentration from platelet lysates. Isolated protein (20 μg) via 4–20% SDS–PAGE was transferred to the PVDF membrane. The primary antibody was treated with a dilution factor of 1:1000 and the secondary antibody was treated with a dilution factor of 1:2000. The results are shown as mean ± SD (*n* = 4). ^a^ *p* < 0.05 in comparison to non−stimulated platelets, * *p* < 0.05, ** *p* < 0.001 in comparison to the U46619−induced platelets.

Artesunate’s effect on PI3K/Akt phosphorylation, a phosphoprotein involved with platelet granule release, was confirmed. As demonstrated in Figure 7A, PI3K and Akt phosphorylation were significantly increased by U46619 in comparison with intact cells. However, the increased PI3K/Akt phosphorylation by U46619 was decreased by artesunate significantly (Figure 7A), demonstrating that artesunate inhibits PI3K/Akt phosphorylation brought on with U46619.

Artesunate’s effect on MAPK (p38, ERK, JNK) phosphorylation, involved in the release of granules in platelets and the production of TXA_2_, was determined. As demonstrated in Figure 7B, p38 phosphorylation was increased significantly by U46619 compared to intact cells, but ERK and JNK phosphorylation was not affected significantly. Further, artesunate significantly inhibited JNK and p38 phosphorylation (Figure 7B). This demonstrates that, via the inhibition of p38 and JNK (MAPK) phosphorylation, the signaling process for the aggregation of platelets was regulated by artesunate.

### 2.7. Effects of Artesunate on Platelet-Mediated Fibrin Clot Retraction

**Figure 8:**
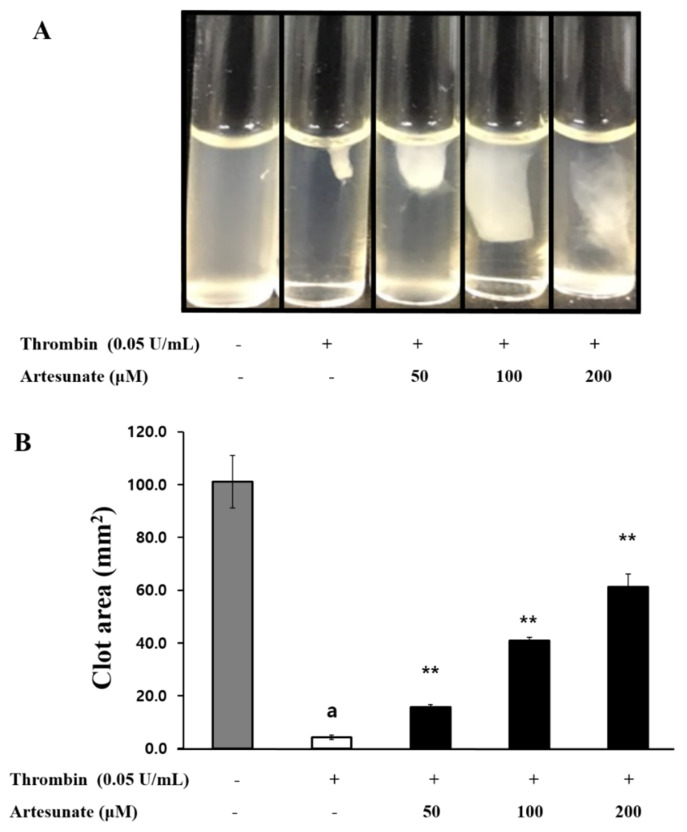
Artesunate’s effect on platelet-mediated fibrin clot retraction. (**A**) Artesunate’s effect on thrombin-retracted fibrin clot photographs. (**B**) Artesunate’s effect on thrombin-retracted fibrin clot area. To avoid sticking, a polyethylene tube contained the PRP (500 μL) and further stimulated via thrombin (0.05 U/mL) and 2 mM CaCl_2_ at a temperature of 37 °C and a time of 15 min. A digital camera then took photographs of the platelets-mediated fibrin clots. The results are shown as mean ± SD (*n* = 4). ^a^ *p* < 0.05 in comparison to non−stimulated platelets, * *p* < 0.05, ** *p* < 0.001 in comparison to the thrombin−induced platelets.

Platelet activation and aggregation occurs through platelet inducers, and over time, signal transduction via the external pathway causes the formation of fibrin clot. Accordingly, the present study investigated artesunate’s effect on platelets-mediated fibrin clot retraction by thrombin induction. As demonstrated in Figure 8A, fibrin clot was strongly formed by thrombin, and artesunate (50, 100, and 200 μM) concentration-dependently inhibited thrombin-induced fibrin clot retraction. The inhibition rates of artesunate (50, 100, and 200 μM) were confirmed to be 11.7%, 37.8%, and 58.8%, respectively (Figure 8B). These results show that artesunate can actually inhibit thrombus formation.

## 3. Discussion

On the platelet membrane, phosphatidylinositol 4,5-bisphosphate (PIP_2_) is hydrolyzed by phospholipase C-γ_2_ (PLC- γ_2_), producing diacylglycerol (DG) and inositol 1,4,5-triphosphate (IP_3_). The IP_3_ from PIP_2_ degradation causes intracellular Ca^2+^ mobilization ([Ca^2+^]_i_) from the dense tubular system into the cytosol and is activated by DG-dependent protein kinase C (PKC) [31]. The increase of [Ca^2+^]_i_ results in myosin light chain and pleckstrin (Ca^2+^/calmodulin dependent proteins) being phosphorylated during platelet aggregation [32].

Having the opposite effect, cyclic nucleotides (cGMP and CAMP) have shown the ability to lower [Ca^2+^]_i_ and inhibit the aggregation of platelets through cGMP-dependent protein kinase (PKG) and cAMP-dependent protein kinase (PKA) [33]. With this study, artesunate significantly increased cAMP production in platelets and caused [Ca^2+^]_i_ inhibition. The results indicate that artesunate-increased cAMP plays a vital part in platelet activation inhibition via down-regulation of [Ca^2+^]_i_. The cAMP, when increased, phosphorylates multiple substrates through the PKA activation, and, more specifically, is known to affect the phosphorylation of inositol 1,4,5-trisphosphate receptor (IP_3_R) [10]. As demonstrated in Figure 3B, artesunate increased concentration-dependent phosphorylation of IP_3_R. This means that the activation of PKA by artesunate caused the phosphorylation of IP_3_R, thereby causing the inhibition of Ca^2+^ channels opening (located in the dense tubular system), thereby reducing [Ca^2+^]_i_. In addition, increased production of cAMP with artesunate led to vasodilator-stimulated phosphoprotein (VASP) being phosphorylated through activation of PKA. VASP is an important substrate of the cAMP/cGMP-dependent kinase (PKA/PKG) that helps with platelet activation regulation through regulating adhesion properties and platelet secretion, and VASP phosphorylation has shown to cause inhibition of the aggregation of platelets via inhibiting activation of integrin αIIb/β_3_ [34,35].

With this study, artesunate strongly inhibited fibrinogen binding to αIIb/β_3_ in platelets induced with U46619. It is thought that the increased cAMP production by artesunate caused the activation of PKA and phosphorylated PKA-dependent VASP Ser157, which caused fibrinogen binding to αIIb/β_3_ to be inhibited. In the future, further research will need to explore the mechanism by which artesunate caused a cAMP production increase. cAMP and cGMP are known to depend on adenylyl cyclase/guanylyl cyclase or cyclic nucleotide phosphodiesterase (PDE) activation [36]. Being that there is an increase in levels of cyclic nucleotides during the aggregation of platelets via PDE activity inhibition, inhibitors of PDE have been shown to have therapeutic effects on thrombosis [37]. As a matter of fact, PDE inhibitors (cilostazol, dipyridamole, and triple rusal) have served as antiplatelet agents to clinically increase cyclic nucleotide production [9]. It is thought that artesunate could be developed as an antiplatelet drug with a comparable function.

In addition, the pathway for PI3K/Akt is noted among phosphoproteins that act during intracellular signaling when platelets are activated, and their phosphorylation plays a major role during platelet function regulation, such as platelet aggregation and dense granule secretion [23]. In addition, mitogen-activated protein kinases (MAPK) are well known as phosphoproteins, including p38 MAPK, c-Jun N-terminal kinase (JNK), and extracellular signal-regulated kinase (ERK), and are involved in the activation and aggregation of platelets [22]. Found in human platelets, MAPKs are shown to have activation via phosphorylation after agonist-activated platelets [17,18]. According to Mei-Chi et al., phosphorylating MAPK (like p38) is critical for arachidonic acid release (a precursor of TXA_2_) and TXA_2_ generation, which leads to the aggregation of platelets [38]. One indicator that is important for the evaluation of substances or components involved in the inhibition of platelet activity is TXA_2_ generation, due to its ability to act as a powerful autacoid which causes additional platelet activation and aggregation. Accordingly, a substance that inhibits TXA_2_ production, such as ozagrel or aspirin, makes for a helpful antiplatelet compound [19,38].

With this study, artesunate showed a significant concentration-dependent inhibi-tory effect on the aggregation of platelets induced by some agonists (U46619, collagen, arachidonic acid), especially by U46619. In particular, in the aggregation induced by U46619, artesunate showed an IC50 of 105.26 µM. However, since this is a result obtained when the washed platelets are treated, there is a limit to explain how much is required when actually administered. Furthermore, considering the case of intranasal administration of artesunate to mice for the treatment of malaria, it can be seen that almost all of it is absorbed and present in the blood for 15 min, but it is reduced by half after 15 min [39]. In fact, when administered to humans, it is expected that a much higher concentration of artesunate will need to be administered, considering that not only the half-life but also the activity of artesunate will be reduced by binding to plasma proteins present in the blood. Nevertheless, this study is significant in clarifying the mechanism through which artesunate exhibits an antiplatelet effect. The effects of artesunate on the production of TXA_2_ and secretion of granules (serotonin and ATP release), which are important markers of platelet aggregation, were measured. Further, we attempted to illuminate artesunate’s relationship with PI3K/Akt and MAPK phosphorylation. As a result, it was established that artesunate suppressed the strongly increased TXA_2_ production with U46619, and the release of serotonin and ATP (intracellular granule secretion indicators) was strongly reduced due to artesunate (Figure 6). Additionally, the results confirmed that PI3K/Akt and MAPK phosphorylation, signaling transduction phosphoproteins noted for granule secretion regulation, was significantly inhibited by artesunate. It is thought that artesunate inhibits phosphoproteins’ (PI3K/Akt and MAPK) phosphorylation, resulting in the aggregation of platelets being inhibited via a reduction in producing TXA_2_ and intracellular secretion of granules (serotonin and ATP release).

**Figure 9:**
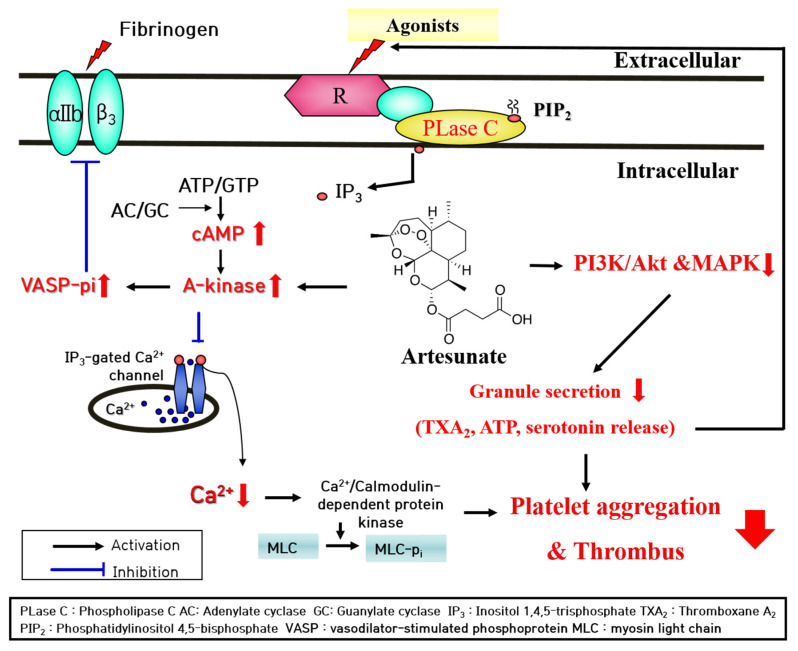
A schematic summary of inhibitory effects of artesunate on the platelet intracellular signaling pathway.

Additionally, increased integrin αIIb/β_3_-mediated signal transduction and granule release generally alter the platelet cytoskeleton, affecting the aggregation of platelets and the formation of a thrombus. Thrombus formation is the utmost vital step while repairing blood vessels that have been damaged, and during this time, activated platelets accumulate and a meshwork of fibrin and platelets develops. When a fibrin clot is forming it begins to contract for 30 to 60 min and ultimately results in a thrombus plug. The retraction of a fibrin clot relies heavily on the fibrinogen and αIIb/β_3_ interaction, and inhibitors of αIIb/β_3_ activity have been shown to prevent the formation of a thrombus [40]. To increase fibrinogen’s ability to bind αIIb/β_3_, coagulation and platelet αIIb/β_3_ activation are induced by thrombin, leading to clot thrombus formation. As demonstrated in Figure 9, artesunate’s antiplatelet effect led to the suppression of thrombin-induced fibrin clots relative to the concentration, with this being the real result of inhibition of thrombus formation. The results here show artesunate has potential as a potent antiplatelet substance that inhibits thrombus formation.

In conclusion, artesunate increased cAMP in human platelets to induce IP_3_R and VASP phosphorylation, which significantly inhibited the recruitment of Ca^2+^ and activation of integrin αIIb/β_3_ into the cytoplasm. In addition, artesunate has been shown to exhibit antiplatelet function by inhibiting granule release by regulating phosphorylation of phosphoproteins acting on signaling transduction such as PI3K/Akt and MAPK. Finally, the production of a thrombin-induced fibrin clot was significantly inhibited. Therefore, we suggest that artesunate has value as a substance that inhibits platelet aggregation and thrombus formation through an antiplatelet mechanism.

## 4. Materials and Methods

### 4.1. Materials

Avention Corporation (Incheon, Korea) provided artesunate (Figure 9). U46619 and thrombin were received from Chrono-Log Corporation (Havertown, PA, USA). The TXB_2_ enzyme immunoassay (EIA) kit, serotonin EIA kit, ATP assay kit, and the cAMP and cGMP EIA kit are available at Cayman Chemical Co. (Ann Arbor, MI, USA). Invitrogen Molecular Probes (Eugene, Orlando, FL, USA) supplied Fibrinogen Alexa Fluor 488 conjugates and Fura 2-AM. Sigma Chemical Corporation (St. Louis, MO, USA) provided the other reagents. Western blotting lysis buffers and antibodies were supplied by Cell Signaling (Beverly, MA, USA). Thermo Fisher Scientific (Seoul, Korea) provided enhanced chemiluminescence solution and polyvinylidene difluoride membrane.

### 4.2. Preparation of Human Washed Platelets

Human platelet-rich plasma (PRP) collected from healthy volunteers, who provided informed consent, was obtained from the Korean Red Cross Blood Center (KRBC, Suwon, Korea), and its experimental use was approved by KRBC and the Namseoul University Institutional Review Board (1041479-HR-201803-003, 23 April 2015). The method previously performed was used to prepare the suspended platelets [41]. For 10 min, PRP was centrifuged at 1300× *g* to collect platelets, then washed (twice) with a buffer (pH 6.9, 2.7 mM KCl, 138 mM NaCl, 12 mM NaHCO_3_, 0.36 mM NaH_2_PO_4_, 1 mM Na_2_EDTA, and 5.5 mM glucose). Suspension buffer (pH 7.4, 2.7 mM KCl, 138 mM NaCl, 12 mM NaHCO_3_, 0.49 mM MgCl_2_, 0.36 mM NaH_2_PO_4_, 5.5 mM glucose, 0.25% gelatin) suspended the platelets (final concentration of 10^8^ cells/mL). Platelet aggregation was avoided by performing all procedures at 25 °C.

### 4.3. Platelet Aggregation Measurement

For 3 min and at 37 °C, incubation of the suspended platelets (10^8^ cells/mL) was carried out while adding artesunate at different concentrations. Then, 2 mM CaCl_2_ and U46619 (0.5 μM) were added for stimulation at a duration of 5 min. The aggregometer (Chrono-Log Co., Havertown, PA, USA) measured the experiment with a stirring speed of 1000 rpm, and the rate of aggregation was calculated with an increase in the light transmittance. The suspension permeability of suspension buffer 0% was used as the reference value. Artesunate was dissolved with a concentration of 0.1% dimethyl sulfoxide (DMSO), and all tests were performed by adding the same dose of DMSO.

### 4.4. Cytotoxicity Measurement

Confirming the release of Lactate dehydrogenase (LDH) from the cytoplasm determined the cytotoxicity. For two hours, incubation of the suspended platelets (10^8^ cells/mL) occurred at room temperature with the addition of different artesunate concentrations, and then it was centrifuged for 2 min at 12,000× *g*. A synergy HT multi-reader (BioTek Instruments, Winooski, VT, USA) measured the supernatants with an LDH EIA kit.

### 4.5. Cyclic Nucleotides (cAMP and cGMP) Production Measurement

For 3 min, incubation of the suspended platelets (10^8^ cells/mL) occurred at 37 °C while adding different artesunate concentrations, then 2 mM CaCl_2_ was added with U46619 (0.5 μM) for stimulation at a duration of 5 min. Termination of the reaction occurred by adding 1 M HCl, and a Synergy HT Multi-Reader (BioTek Instruments, Winooski, VT, USA) measured cGMP/cAMP using the cAMP or cGMP EIA kit.

### 4.6. Intracellular Ca^2+^ Mobilization Measurement

For 60 min, incubation of PRP occurred with Fura 2-AM (5 μM) at 37 °C, and the suspended platelets (10^8^ cells/mL) were prepared based on the steps specified above. The incubation of washed platelets occurred for 3 min at 37 °C with 2 mM CaCl_2_ and U46619 (0.5 μM) stimulation for a duration of 5 min. A spectrofluorometer (SFM 25, USA) from BioTeck Instrument (Winooski, VT, USA) measured the Fura 2 fluorescence. The fluorescence was analyzed with excitation wavelengths at 340 and 380 nm and emission at 510 nm. The calculation of the amount of Ca^2+^ mobilized was performed using the method of Grynkiewicz [42].

### 4.7. Fibrinogen Binding Measurement

After treatment with Alexa Fluor 488-human fibrinogen (30 μg/mL) by adding 2 mM CaCl_2_ to the suspended platelets (10^8^ cells/mL), U46619 (0.5 μM) was used for stimulation at a duration of 5 min. The addition of phosphate-buffered saline (PBS, pH 7.4) containing 0.5% paraformaldehyde terminated the reaction. Light blocking was used during this process and a flow cytometer (FACS) from BD Bioscience (San Jose, CA, USA) measured fibrinogen binding. The software, Cell-Quest (BD Biosciences), was used to analyze.

### 4.8. TXB_2_ Production Measurement

For 3 min, incubation of the suspended platelets (10^8^ cells/mL) occurred at 37 °C while adding different artesunate concentrations, then 2 mM CaCl_2_ was added with U46619 (0.5 μM) for stimulation at a duration of 5 min. A synergy HT multi-reader (BioTek Instruments, Winooski, VT, USA) measured TXB_2_ (a stable TXA_2_ metabolite) production with the use of the TXB_2_ EIA kit.

### 4.9. ATP and Serotonin Release Measurement

For 3 min, incubation of the suspended platelets (10^8^ cells/mL) occurred at 37° with different artesunate concentrations, then 2 mM CaCl_2_ were added with U46619 (0.5 μM) for stimulation at a duration of 5 min. Once the reaction was stopped with ice-cold 2 mM EDTA, a synergy HT multi-reader (BioTek Instruments, Winooski, VT, USA) and serotonin or ATP EIA kit measured the released serotonin/ATP in the upper layer due to centrifuging. The degree of secretion of granular substances in platelets out of the cells was confirmed by measuring ATP or serotonin in the upper layer by centrifugation after an aggregation reaction.

### 4.10. Western Immunoblotting Measurement

Termination of the reaction was carried out using a 1× lysis buffer. A BCA protein kit (Pierce Biotechnology, Rockford, IL, USA) was used to measure the concentration of proteins from platelet lysates. Protein (20 μg) was isolated via 4–20% SDS–PAGE and transferred to PVDF membrane. The primary antibody was treated with a dilution factor of 1:1000 and the secondary antibody was treated with a dilution factor of 1:2000. Visualization was performed using ECL reagent (Thermo Fisher Scientific, Gangnam-gu, Seoul, Korea).

### 4.11. Platelet-Mediated Fibrin Clot Retraction Measurement

To avoid sticking, a polyethylene tube contained the PRP (500 μL) which was then stimulated with thrombin (0.05 U/mL) and 2 mM CaCl_2_ at a temperature of 37 °C and a time of 15 min. A digital camera then took photographs of the platelets-mediated fibrin clots. The software, ImageJ (version 1.46, National Institutes of Health, Bethesda, MD, USA), calculated the area of coagulation.

### 4.12. Statistical Analysis

All data are presented as mean ± standard deviation with varying number of observations. A repeated experiment was performed with platelets from four normal adults. ANOVA was performed to identify major differences between groups, and Scheffe’s method was used. Statistical analysis was performed using SPSS 21.0.0.0 software (SPSS, Chicago, IL, USA), and *p* < 0.05 was considered statistically significant.

## Data Availability

Not applicable.
